# Efficient Localization Method Based on RSSI for AP Clusters

**DOI:** 10.3390/s23177599

**Published:** 2023-09-01

**Authors:** Zhigang Su, Zeyu Tian, Jingtang Hao

**Affiliations:** Sino-European Institute of Aviation Engineering, Civil Aviation University of China, Tianjin 300300, China; tian_tzy@163.com (Z.T.); jthao_siae@126.com (J.H.)

**Keywords:** indoor localization, received signal strength indication, maximum likelihood estimation, access point clusters, eigenvalue method, real-time localization

## Abstract

The localization accuracy is susceptible to the received signal strength indication (RSSI) fluctuations for RSSI-based wireless localization methods. Moreover, the maximum likelihood estimation (MLE) of the target location is nonconvex, and locating target presents a significant computational complexity. In this paper, an RSSI-based access point cluster localization (APCL) method is proposed for locating a moving target. Multiple location-constrained access points (APs) are used in the APCL method to form an AP cluster as an anchor node (AN) in the wireless sensor network (WSN), and the RSSI of the target is estimated with several RSSI samples obtained by the AN. With the estimated RSSI for each AN, the solution for the target location can be obtained quickly and accurately due to the fact that the MLE localization problem is transformed into an eigenvalue problem by constructing an eigenvalue equation. Simulation and experimental results show that the APCL method can meet the requirement of high-precision real-time localization of moving targets in WSN with higher localization accuracy and lower computational effort compared to the existing classical RSSI-based localization methods.

## 1. Introduction

In recent years, location-based services related to daily life and work have been launched [1]. For large indoor areas, such as factories, hospitals, and shopping malls, the ability to locate moving targets inside them in real time is necessary for purposes such as navigation, surveillance, and business model optimization [2]. The commonly employed techniques for indoor wireless localization techniques encompass Wi-Fi, radio frequency identification (RFID), ultra-wideband (UWB), long-range radio (LoRa), bluetooth low energy (BLE), and ZigBee, etc. [3,4,5,6]. The increasing density of Wi-Fi coverage and the widespread adoption of mobile devices equipped with wireless network interface controllers have significantly enhanced the ubiquity of Wi-Fi-based localization technologies, eliminating the need for additional hardware. For Wi-Fi-based localization technology, the wireless access points (APs) are used as the anchor nodes (ANs) to form a wireless sensor network (WSN), and the target is considered as the blind node. The AP measures the radio signal parameters emitted by the target for localization, such as time of arrival (TOA) [7], time difference of arrival (TDOA) [8], angle of arrival (AOA) [9], received signal strength indication (RSSI) [10], etc. Measuring the target RSSI requires neither clock synchronization nor antenna arrays, and has a lower cost, so RSSI-based localization methods are more promising for application and have received wide attention from scholars [11].

The RSSI-based localization problem consists in the need to obtain the distance information between the target and AN using the target RSSI samples measured by the ANs, and then obtain the target location estimate with the distance information. The essence of this localization problem is the obtention of the maximum likelihood estimation (MLE) of the target location [12,13]. However, this problem is a nonconvex optimization problem with multiple locally optimal solutions. Moreover, the complexity of the indoor Wi-Fi channel leads to drastic fluctuations in the RSSI measured by the ANs, which leads to a reduction in the accuracy of the traditional iterative methods for solving the target location. The search for ways in which RSSI can be effectively used to localize target with superior accuracy has become a hot issue in the field of indoor localization.

To improve the accuracy of the RSSI-based localization, it is necessary to improve the accuracy of the RSSI for the target characterized by AN. RSSI samples measured by the AN are processed, such as mean filter [14], Kalman filter [15], Gaussian filter [16], etc., to reduce the influence of random factors and improve the accuracy of the target RSSI. However, existing methods for treating RSSI do not consider the restriction on the number of RSSI samples. During real-time localization, the number of RSSI samples measured by AN is mostly limited due to the limited frequency of RSSI measurements, which leads to degraded performance of these methods.

For non-convexity of the RSSI-based localization problem, commonly used methods include the convex relaxation method [17,18,19,20,21] and objective function approximation method [22,23]. The convex relaxation method transforms a nonconvex optimization problem into a convex optimization problem by relaxing the constraints to find a globally optimal solution. Typical convex relaxation methods contain the semidefinite programming (SDP) algorithm [17], the second-order cone programming (SOCP) algorithm [20,21], etc. Despite the better accuracy, the computational complexity of the convex relaxation method is significant. The objective function approximation method approximates the original problem in order to find the global optimal solution of the approximated problem. Typical methods include weighted least squares (WLS) [22], squared range least squares (SRLS) [23], etc. Although the objective function approximation method has a relatively simple computational procedure, the approximation procedure introduces additional errors that lead to lower localization accuracy. Existing methods for solving the non-convexity of localization problems fail to balance accuracy and computational complexity at the same time, thus failing to meet the demand for high-precision real-time localization.

To address the localization error and the computational cost caused by RSSI fluctuation and non-convexity of the localization problem during real-time localization, in this paper, an RSSI-based AP cluster localization (APCL) method is proposed for high-precision real-time localization of indoor mobile targets. First, the AP cluster is proposed to form the AN in order to increase the number of RSSI samples measured by a single AN. Then, the optimal RSSI of the target is estimated from the samples measured by AN to reduce the error due to fluctuations. Finally, the MLE problem is constructed as an eigenvalue problem and the global optimal solution can be solved directly and quickly to reduce the error and computational complexity due to non-convexity. The main contributions of this paper are as follows.

(1) It is proposed to construct the AN in the form of an AP cluster, and use the AP cluster to obtain multiple RSSI samples of a single AN. The proposed target RSSI estimation method is based on a limited number of RSSI samples, and the optimal RSSI estimation is beneficial to improve the target localization accuracy.
(2) A method is proposed to transform the RSSI-based localization problem into an eigenvalue problem, which can well solve the nonconvex problem and obtain the global optimal solution with great accuracy and low computational complexity.

The rest of the paper is organized as follows: In Section 2, the problem studied in this paper is described. Theoretical analysis on utilizing multiple APs for establishing an AN is presented in Section 3. The method presented in Section 4 is used to estimate the optimal RSSI of a target using samples from AP cluster measurements. In Section 5, a way to transform the MLE problem into an eigenvalue problem is described. The proposed APCL method is summarized in Section 6. The Cramer–Rao lower bound (CRLB) of the localization method which utilizes the AP cluster to form the AN is analyzed in Section 7. The computational complexity of the APCL method is analyzed in Section 8. Several simulations and experimental results are discussed in Section 9 and Section 10, respectively. Finally, some conclusions are given for the paper in Section 11. Key notations are given in Table 1.

## 2. Problem Statement

**Figure 1:**
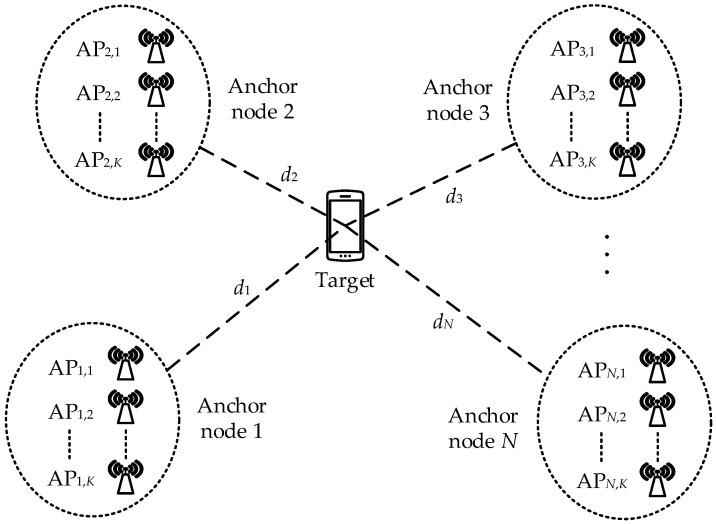
Schematic diagram of AP clusters measuring target RSSI.

We consider a target in a WSN comprising *N* ANs, where each AN is composed of a cluster of *K* APs located in close proximity to each other, as depicted in Figure 1. The location of the *n*th AN within the WSN is known and represented by position vector $s_{n}={\left[x_{n},y_{n}\right]}^{T}$. On the other hand, position vector $u={\left[x,y\right]}^{T}$ indicates the unknown position of the target. Consequently, we can express the distance between the target and the *n*th AN as

(1) $$d_{n}=∥u-s_{n}∥,$$

where $∥\cdot ∥$ is the Frobenius norm. It is evident that the localization system depicted in Figure 1 conforms to a conventional WSN localization system when K=1.

Performance discrepancies among APs in a WSN can be rectified through device calibration during the initial setup, thereby ensuring uniform performance across all APs within the network. The received RSSI of a target detected by the *k*th AP in the *n*th AN is denoted as $r_{n,k}$. Based on the log-normal model for RSSI measurements [24], $r_{n,k}$ can be mathematically expressed as

(2) $$r_{n,k}=r_{0}-10\eta \ln\left(d_{n}\right)+\epsilon_{n,k},\;k=1,2,\cdots ,K,$$

where $r_{0}$ represents the RSSI of the target, which is measured by the AP at a distance of 1 m in an ideal environment. The symbol η denotes the path loss exponent in the localization environment. Additionally, $\epsilon_{n,k}$ signifies the shadow fading term for the *k*th AP in the *n*th AN. Significantly, these shadow fading terms at different APs are commonly modeled as independent and identically distributed Gaussian random variables with zero mean and variance $\sigma^{2}$ [17,18,19,20].

For the *n*th AN, the *K* APs within it are capable of obtaining *K* RSSI measurements from the target, which collectively form the set of RSSI samples measured by this AN:

(3) $$S_{n}=\left\{r_{n,1},r_{n,2},\cdots ,r_{n,K}\right\}.$$

Using the samples in $S_{n}$, an appropriate method can be employed to obtain the optimal estimate ${\hat{r}}_{n,\mathrm{opt}}$ of the target RSSI for the *n*th AN. By substituting ${\hat{r}}_{n,\mathrm{opt}}$ into Equation (2), it is possible to estimate the distance between the *n*th AN and the target

(4) $${\hat{d}}_{n}=\exp\left\{\left(r_{0}-{\hat{r}}_{n,\mathrm{opt}}\right)/10\eta \right\}.$$

Based on the optimal estimates of the target RSSI for *N* ANs, the likelihood function regarding the target location can be derived from the model presented in Equation (2):

(5) $$L\left({\hat{r}}_{1,\mathrm{opt}},{\hat{r}}_{2,\mathrm{opt}},\cdots ,{\hat{r}}_{N,\mathrm{opt}}|u\right)=\prod_{n=1}^{N}\frac{1}{\sqrt{2\pi }\sigma }\exp\left\{-\frac{1}{2\sigma^{2}}{\left({\hat{r}}_{n,\mathrm{opt}}-r_{0}+10\eta \ln d_{n}\right)}^{2}\right\}.$$

By utilizing Equations (4) and (5), it can be further simplified as

(6) $$L\left({\hat{r}}_{1,\mathrm{opt}},{\hat{r}}_{2,\mathrm{opt}},\cdots ,{\hat{r}}_{N,\mathrm{opt}}|u\right)=\prod_{n=1}^{N}\frac{1}{\sqrt{2\pi }\sigma }\exp\left\{-\frac{{\left(10\eta \right)}^{2}}{2\sigma^{2}}{\left(\ln d_{n}-\ln{\hat{d}}_{n}\right)}^{2}\right\}.$$

According to Equation (1), it can be inferred that $d_{n}$ is a function of u. Therefore, the maximization of the likelihood function presented in Equation (6) is equivalent to the minimization the cost function with respect to u:

(7) $$C\left(u\right)=\sum_{n=1}^{N}{\left(\ln d_{n}^{2}-\ln{\hat{d}}_{n}^{2}\right)}^{2}.$$

For the purpose of enhancing the subsequent cost function $C\left(u\right)$ minimization, we opt for $d_{n}^{2}$ over $d_{n}$ due to its superior convenience. Evidently, the target location estimation can be achieved by efficiently minimizing $C\left(u\right)$:

(8) $$\hat{u}=\arg\min_{u}C\left(u\right).$$

As observed from the aforementioned analysis, there are three issues that need to be addressed in order to acquire the location estimate of the target: first, constructing an AP cluster in which multiple APs function collectively as a unified AN; second, determining the optimal RSSI estimate ${\hat{r}}_{n,\mathrm{opt}}$ of the target based on $R_{n}$; finally, minimizing the cost function $C\left(u\right)$ to obtain the maximum likelihood estimate $\hat{u}$ of the target location.

## 3. Establishing an AN with Multiple APs

**Figure 2:**
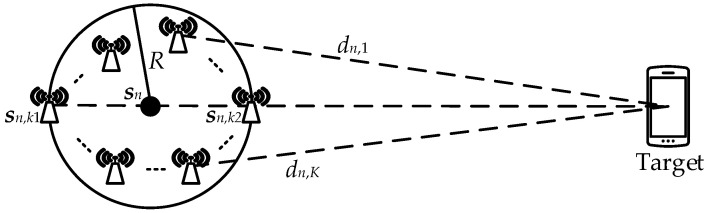
Schematic diagram of the formation of an AP cluster in AN.

The *K* APs comprising an AN are assumed to be positioned in a circular arrangement with a radius of *R*, centered at the location of AN, as shown in Figure 2. In the graph, the position vector of the *k*th AP is $s_{n,k}={\left[x_{n,k},y_{n,k}\right]}^{T}$, and $d_{n,k}$ represents the distance between the target and the *k*th AP. It can be seen from the geometric relationship in the graph that for any two $d_{n,k1}$ and $d_{n,k2}$, there exists

(9) $$\left|d_{n,k1}-d_{n,k2}\right|\leq 2R.$$

The equality in Equation (9) holds when the *k*1th and *k*2th APs are positioned on the straight line connecting AN and the target, with separate circles on both sides of AN. The current layout of the two APs is evidently the worst. Assuming $d_{n,k1}>d_{n,k2}$, the RSSI from both APs satisfies $r_{n,k1}>r_{n,k2}$. Disregarding noise, Equation (2) can be utilized to derive this relationship:

(10) $$r_{n,k2}-r_{n,k1}=10\eta \ln\frac{d_{n,k1}}{d_{n,k2}}=10\eta \ln\frac{d_{n}+d_{n,k1}-d_{n}}{d_{n}-d_{n}+d_{n,k2}}=10\eta \ln\frac{d_{n}+R}{d_{n}-R}.$$

The measurement accuracy of RSSI by AP in practical is typically 1 dBm. Therefore, the two APs are considered to belong to the same AN when the difference between the theoretical measurements of $r_{n,k1}$ and $r_{n,k2}$ is less than 1 dBm. Hence,

(11) $$10\eta \ln\frac{d_{n}+R}{d_{n}-R}\leq 1.$$

It can be obtained from Equation (11) that

(12) $$R_{\max}=\frac{\exp\left\{1/10\eta \right\}-1}{\exp\left\{1/10\eta \right\}+1}d_{n}.$$

The value of the maximum radius $R_{\max}$ of the smallest circle surrounding the AP cluster is evidently influenced by both distance $d_{n}$ between the target and AN and environmental parameter η. The constraint on the radius of the AP cluster is relatively relaxed for targets located at a distance. The layout of WSN in indoor positioning applications typically exceeds 10 m, and the distance between the target and the sensor is seldom less than 1 m. Hence, $d_{n}$ in Equation (12) may have a value of 1 m. The value range of environmental parameter η was analyzed and provided in [25]. Under the condition of natural logarithm, η≤2.6. Consequently, Equation (12) yields $R_{\max}=2$ cm.

The antennas of the multiple APs that constitute the AP cluster should be strategically positioned in a circular arrangement with a radius not exceeding $R_{\max}$. The center of the circle corresponds to the AN position.

## 4. Estimating the Optimal RSSI of a Target

According to the log-normal model presented in Equation (2), there exists an approximate logarithmic relationship between the RSSI measurement error and the ranging error, resulting in a wider range of variation in the ranging errors compared to RSSI measurement errors. To enhance target localization accuracy, the primary task is to improve the precision of RSSI used for ranging by preprocessing AN-measured RSSI samples to obtain the optimal RSSI estimate of target [26].

Since the shadow fading term, which is responsible for RSSI fluctuations, follows a zero-mean Gaussian distribution, the optimal estimate ${\hat{r}}_{n,\mathrm{opt}}$ of the target RSSI for the *n*th AN can be obtained by the following equation:

(13) $${\hat{r}}_{n,\mathrm{opt}}=\arg\min_{r_{n}}\sum_{k=1}^{K}{\left(r_{n,k}-r_{n}\right)}^{2}.$$

Then, the solution of Equation (13) is the sample mean ${\bar{r}}_{n}$ of $S_{n}$:

(14) $${\hat{r}}_{n,\mathrm{opt}}={\bar{r}}_{n}.$$

Obviously, the accuracy of ${\hat{r}}_{n,\mathrm{opt}}$ is closely related to *K*. When *K* reaches a certain threshold, highly accurate results for ${\hat{r}}_{n,\mathrm{opt}}$ can be yielded from Equation (14). However, due to cost considerations, typically only two or three APs are deployed by a single AN at a time. In such cases, the use of sample mean ${\bar{r}}_{n}$ as ${\hat{r}}_{n,\mathrm{opt}}$ can lead to significant error, and it thus necessitates the development of a new method for determining ${\hat{r}}_{n,\mathrm{opt}}$.

Replacing ${\hat{r}}_{n,\mathrm{opt}}$ in Equation (4) with the sample $r_{n,k}$ in $S_{n}$ yields the corresponding distance estimate ${\hat{d}}_{n,k}$. It is evident that a more reliable estimate can be obtained by utilizing a larger $r_{n,k}$ resulting in a smaller ${\hat{d}}_{n,k}$. Therefore, employing the distance estimates to define weights

(15) $$\gamma_{n,k}=\left\{\begin{array}{cc} 2-\frac{{\hat{d}}_{n,k}}{{\bar{d}}_{n}}, & {\hat{d}}_{n,k}<{\bar{d}}_{n}\\ \frac{{\bar{d}}_{n}}{{\hat{d}}_{n,k}}, & {\hat{d}}_{n,k}\geq {\bar{d}}_{n}, \end{array}\right.$$

where ${\bar{d}}_{n}$ represents the estimated distance obtained by substituting ${\hat{r}}_{n,\mathrm{opt}}$ in Equation (4) with the sample mean ${\bar{r}}_{n}$. By incorporating the weights illustrated in Equations (15), Equations (14) can be adjusted as

(16) $${\hat{r}}_{n,\mathrm{opt}}=\frac{1}{\gamma_{n}}\sum_{k=1}^{K}\gamma_{n,k}r_{n,k},$$

where $\gamma_{n}$ is utilized for weight normalization, representing the cumulative sum of weights assigned to all APs within the *n*th AN, denoted as $\gamma_{n}=\sum_{k=1}^{K}\gamma_{n,k}$.

## 5. Revising the MLE for Determining the Location of the Target

Performing a first-order Taylor expansion of $\ln d_{n}^{2}$ in the cost function $C\left(u\right)$ at ${\hat{d}}_{n}^{2}$ yields

(17) $$\ln d_{n}^{2}=\ln{\hat{d}}_{n}^{2}+\frac{1}{{\hat{d}}_{n}^{2}}\left(d_{n}^{2}-{\hat{d}}_{n}^{2}\right).$$

Substituting Equation (17) and Equation (1) into Equation (7), we obtain the following expression:

(18) $$C\left(u\right)=\sum_{n=1}^{N}\frac{1}{{\hat{d}}_{n}^{4}}{\left({∥u-s_{n}∥}^{2}-{\hat{d}}_{n}^{2}\right)}^{2}.$$

It can be observed that cost function $C\left(u\right)$ is a weighted aggregation of the error functions of the ANs where $1/{\hat{d}}_{n}^{4}$ serves as the weight factor. The process of weight normalization is executed:

(19) $$w_{n}=\frac{1/{\hat{d}}_{n}^{4}}{w},$$

where $w=\sum_{n=1}^{N}1/{\hat{d}}_{n}^{4}$ is utilized for weight normalization. Subsequently, the cost function $C\left(u\right)$ can be updated as

(20) $$C\left(u\right)=\sum_{n=1}^{N}w_{n}{\left({∥u-s_{n}∥}^{2}-{\hat{d}}_{n}^{2}\right)}^{2}.$$

Minimizing $C\left(u\right)$ is equivalent to determining u that yields a first-order derivative of $C\left(u\right)$ equal to zero, thereby solving the following equation:

(21) $$\nabla C\left(u\right)=4\sum_{n=1}^{N}w_{n}\left(u^{T}u-2u^{T}s_{n}+s_{n}^{T}s_{n}-{\hat{d}}_{n}^{2}\right)\left(u-s_{n}\right)=0.$$

Assuming $s_{w}=\sum_{n=1}^{N}w_{n}s_{n}$, $s_{w}$ represents the weighted location vector of *N* AN’s coordinates. Relocating the coordinate origin to $s_{w}$ yields

(22) $$\left\{\begin{array}{c} u^{\prime }=u-s_{w}\\ s_{n}^{\prime }=s_{n}-s_{w} \end{array}\right..$$

Therefore, we have $\sum_{n=1}^{N}w_{n}s_{n}^{\prime }=0$. By substituting Equation (22) into Equation (21), we obtain

(23) $$\sum_{n=1}^{N}w_{n}\left(u^{{}^{\prime }T}u^{\prime }-2u^{{}^{\prime }T}s_{n}^{\prime }+s_{n}^{{}^{\prime }T}s_{n}^{\prime }-{\hat{d}}_{n}^{2}\right)\left(u^{\prime }-s_{n}^{\prime }\right)=0.$$

Consequently, we have

(24) $$\left(u^{{}^{\prime }T}u^{\prime }\right)u^{\prime }+Au^{\prime }+b^{\prime }=0,$$

where

(25) $$A=\sum_{n=1}^{N}\left\{w_{n}\left[2s_{n}^{\prime }s_{n}^{{}^{\prime }T}+\left(s_{n}^{{}^{\prime }T}s_{n}^{\prime }-{\hat{d}}_{n}^{2}\right)I_{2}\right]\right\}$$

and

(26) $$b^{\prime }=\sum_{n=1}^{N}\left\{w_{n}\left({\hat{d}}_{n}^{2}-s_{n}^{{}^{\prime }T}s_{n}^{\prime }\right)s_{n}^{\prime }\right\},$$

where $I_{2}$ is the second-order identity matrix.

From Equation (25), it is evident that $A=A^{T}$ holds, thus enabling the possibility of performing an eigenvalue decomposition on A:

(27) $$A=UDU^{T},$$

where D is a diagonal matrix composed of the eigenvalues obtained from A, U is a unitary matrix consisting of the eigenvectors derived from A. Considering $v=U^{T}u^{\prime }$ and $b=U^{T}b^{\prime }$, we can deduce $u^{\prime }=Uv$ and $b^{\prime }=Ub$ accordingly. By substituting $u^{\prime }=Uv$, $b^{\prime }=Ub$, and Equation (27) into Equation (24), followed by pre-multiplying with $U^{T}$, we obtain

(28) $$\left(v^{T}v\right)v=-Dv-b.$$

By further performing the Hadamard product of Equation (28) with v, we obtain

(29) $$\left(v^{T}v\right)⊙v=-Dv⊙v-\mathrm{diag}\left\{b\right\}v,$$

where ⊙ denotes the Hadamard product operation, and $\mathrm{diag}\left\{\cdot \right\}$ denotes the transformation of a column vector into a diagonal matrix. Therefore, we obtain

(30) $$v^{T}v=1^{T}\left(v⊙v\right),$$

where ***1*** denotes a two-dimensional column vector consisting of all elements equal to one.

By integrating Equations (28)–(30), a matrix is formulated:

(31) Mz=λz,

where $\lambda =\left(v^{T}v\right)$, and M is a 5 × 5 matrix that can be represented as

(32) $$M=\left[\begin{array}{ccc} -D & -\mathrm{diag}\left\{b\right\} & 0_{2\times 1}\\ 0_{2\times 2} & -D & -b\\ 1^{T} & 0_{1\times 2} & 0 \end{array}\right],$$

where $0_{i\times k}$ is a matrix of size i×k, consisting entirely of zero elements, $z={\left[v⊙v,v,1\right]}^{T}$ is the eigenvector of M, and $\lambda =v^{T}v$ corresponds to the eigenvalue associated with z. As per Equation (30), we can infer that λ equals the sum of the first two elements in z.

The eigenvalue decomposition of M yields

(33) $$M=U_{M}D_{M}{U_{M}}^{-1},$$

where $D_{M}$ is a diagonal matrix composed of $\lambda_{m}\left(m=1,\cdots ,5\right)$, which represent the eigenvalues of M, $U_{M}$ is the matrix consisting of $z_{m}$, which are the corresponding eigenvectors of M and are normalized. However, since the sum of the first two elements in each row of $z_{m}$ does not equal to $\lambda_{m}$, it cannot be directly used to extract $v_{m}$. Therefore, scaling $z_{m}$ with $\lambda_{m}$ and adjusting for the sum of its first two elements is necessary before extracting the third and fourth elements as an estimate for $v_{m}$:

(34) $${\hat{v}}_{m}=\frac{\lambda_{m}}{e^{T}z_{m}}\left[0_{2\times 2},I_{2},0_{2\times 1}\right]z_{m},$$

where $e={\left[1,1,0,0,0\right]}^{T}$. Based on this, it is possible to derive an estimate of the potential target location:

(35) $${\hat{u}}_{m}=U{\hat{v}}_{m}+s_{w}.$$

With varying $z_{m}$, distinct ${\hat{u}}_{m}$ can be obtained and subsequently substituted into Equation (20) to compute the values of the cost function $C\left(u\right)$. The ${\hat{u}}_{m}$ that minimizes $C\left(u\right)$ is then selected as the estimate for the target location.

## 6. APCL Method

As mentioned above, the APCL method proposed in this paper comprises two relatively independent components: one aims to achieve the optimal RSSI estimation of the target by utilizing the RSSI measurements obtained from each AP in the AN, and the other focuses on solving the maximum likelihood estimate of the target location within the WSN.

The algorithm for estimating the optimal RSSI from an AN to a target is known as the relative distance weighting (RDW) algorithm, which is summarized in Algorithm Section 6. For the *n*th AN, the RDW algorithm first uses all the RSSI samples ${\left\{r_{n,k}\right\}}_{k=1}^{K}$ measured by this AN and the sample mean ${\bar{r}}_{n}$ to calculate the corresponding distance estimates ${\left\{{\hat{d}}_{n,k}\right\}}_{k=1}^{K}$ and ${\bar{d}}_{n}$ based on the log-normal model. Then, weights ${\left\{\gamma_{n,k}\right\}}_{k=1}^{K}$ are assigned to each sample according to their respective distances. Finally, the optimal estimate of target RSSI is obtained by normalizing the weighted sum of all RSSI samples, as demonstrated in Equation (16). By traversing all ANs, we can obtain ${\left\{{\hat{r}}_{n,\mathrm{opt}}\right\}}_{n=1}^{N}$.

**Algorithm 1** Relative distance weighting for estimating the optimal RSSI of the target. **Input:**
$r_{0}$ and η, parameters of the log-normal model, and ${\left\{S_{n}\right\}}_{n=1}^{N}$, a set of RSSI samples measured by each AN **Output:**
${\left\{{\hat{r}}_{n,\mathrm{opt}}\right\}}_{n=1}^{N}$, a set of optimal estimates of the target RSSI for each AN1: **for**
n=1,2,⋯,N **do**2: Calculate the sample mean ${\bar{r}}_{n}$ and its corresponding distance estimate ${\bar{d}}_{n}$ for the *n*-th AN;3: Refine the distance estimates ${\left\{{\hat{d}}_{n,k}\right\}}_{k=1}^{K}$ for samples ${\left\{r_{n,k}\right\}}_{k=1}^{K}$ in the *n*th AN;4: The weights ${\left\{\gamma_{n,k}\right\}}_{k=1}^{K}$ corresponding to all APs within the *n*th AN can be obtained by substituting ${\left\{{\hat{d}}_{n,k}\right\}}_{k=1}^{K}$ and ${\bar{d}}_{n}$ into Equation (15);5: Calculate the sum of ${\left\{\gamma_{n,k}\right\}}_{k=1}^{K}$ to obtain $\gamma_{n}$;6: Substitute ${\left\{\gamma_{n,k}\right\}}_{k=1}^{K}$, $\gamma_{n}$ and ${\left\{r_{n,k}\right\}}_{k=1}^{K}$ into Equation (16) to obtain the optimal estimate of the target RSSI for the *n*th AN.7: **end for**8: **return**
${\left\{{\hat{r}}_{n,\mathrm{opt}}\right\}}_{n=1}^{N}$

After obtaining the optimal RSSI estimate of the target for each AN, an eigenvector-based target localization (ETL) algorithm is proposed in this paper to obtain the maximum likelihood estimate of the target location. The algorithm is summarized in Algorithm Section 6. The first two steps of the ETL algorithm constitute the initialization phase. According to ${\left\{{\hat{r}}_{n,\mathrm{opt}}\right\}}_{n=1}^{N}$, the distance estimates ${\left\{{\hat{d}}_{n}\right\}}_{n=1}^{N}$ between each AN and the target can be calculated in order to obtain the normalized weights ${\left\{w_{n}\right\}}_{n=1}^{N}$ for each AN. Steps 3 to 8 represent the second phase of the ETL algorithm, with its main objective being the construction of data matrix M. To simplify the process of minimizing cost function $C\left(u\right)$ as depicted in Equation (20), a weighted location vector is employed. By translating the coordinate system, we transform the problem of minimizing $C\left(u\right)$ into that shown in Equation (24). Subsequently, we perform variable substitution on Equation (24) using U to obtain Equation (28). Based on Equations (28), Equation (31) is constructed using the Hadamard product. The data matrix M is constructed with the eigenvalues matrix D and the vector b as in Equation (32). The final phase of the ETL algorithm involves obtaining the corresponding eigenvalue matrix $D_{M}$ and the eigenvector matrix $U_{M}$ through the eigenvalue decomposition of M. Each pair of eigenvalue $\lambda_{m}$ and eigenvector $z_{m}$ can be used to derive a potential target location estimate ${\hat{u}}_{m}$. The ${\hat{u}}_{m}$ that minimizes $C\left(u\right)$ is selected as the estimated target location $\hat{u}$.

**Algorithm 2** Eigenvector-based target localization algorithm. **Input:**
$r_{0}$ and η, parameters of the log-normal model, ${\left\{s_{n}\right\}}_{n=1}^{N}$, a set of location vectors for each AN, and ${\left\{{\hat{r}}_{n,\mathrm{opt}}\right\}}_{n=1}^{N}$, a set of optimal target RSSI estimates for each AN **Output:**
$\hat{u}$, the maximum likelihood estimate of the target location ∖∗Initialization∗∖1: Substitute ${\left\{{\hat{r}}_{n,\mathrm{opt}}\right\}}_{n=1}^{N}$ into Equation (4) to calculate the estimated distance ${\left\{{\hat{d}}_{n}\right\}}_{n=1}^{N}$ between each AN and the target;2: Sum ${\left\{1/{\hat{d}}_{n}^{4}\right\}}_{n=1}^{N}$ to obtain *w*, and then calculate the normalized weights ${\left\{w_{n}\right\}}_{n=1}^{N}$ for all ANs from Equation (19);∖∗Constructing the data matrix∗∖3: Calculate the weighted location vector $s_{w}=\sum_{n=1}^{N}w_{n}s_{n}$ of *N* ANs;4: Based on vector $s_{w}$, the new location vector of each AN ${\left\{s_{n}^{\prime }\right\}}_{n=1}^{N}$ can be obtained from Equation (22) after translating the coordinate system;5: Construct the matrix A and the vector $b^{\prime }$ according to Equations (25) and (26), respectively;6: Perform the eigenvalue decomposition of matrix A to obtain the diagonal matrix D consisting of its eigenvalues and the unitary matrix U composed of its eigenvectors;7: Transform the vector $b^{\prime }$ to the vector $b=U^{T}b^{\prime }$ by using U;8: Substitute D and b into Equation (32) to construct the data matrix M;∖∗Estimation of target location using eigenvalues∗∖9: Perform the eigenvalue decomposition of M to obtain the diagonal matrix $D_{M}$ consisting of the eigenvalues ${\left\{\lambda_{m}\right\}}_{m=1}^{5}$ and the matrix $U_{M}$ composed of the corresponding eigenvectors ${\left\{z_{m}\right\}}_{m=1}^{5}$;10: Apply Equation (34) to scale $z_{m}$ using $\lambda_{m}$, and then use Equation (35) to calculate the estimated potential location of the target, denoted as ${\hat{u}}_{m}$;11: By substituting ${\hat{u}}_{m}$ into Equation (20) to calculating the cost function $C\left({\hat{u}}_{m}\right)$, the ${\hat{u}}_{m}$ that minimizes $C\left({\hat{u}}_{m}\right)$ is utilized as the estimate for the target location, denoted by $\hat{u}={\hat{u}}_{m}$.12: **return**
$\hat{u}$

Hence, the APCL method initially employs the RDW algorithm to estimate the optimal RSSIs ${\left\{{\hat{r}}_{n,\mathrm{opt}}\right\}}_{n=1}^{N}$ of the target for all ANs based on the set of RSSI samples ${\left\{S_{n}\right\}}_{n=1}^{N}$. Subsequently, it utilizes the ETL algorithm to derive the maximum likelihood estimate $\hat{u}$ of the target location by integrating the location vector information ${\left\{s_{n}\right\}}_{n=1}^{N}$ of multiple ANs.

The source codes are available for download from: https://blog.csdn.net/weixin_42428226/article/details/132175267?spm=1001.2014.3001.5501 (accessed on 9 August 2023).

## 7. Analysis of Cramer–Rao Lower Bound

For a WSN comprising *N* ANs, each containing *K* APs, based on the log-normal model presented in Equation (2), the likelihood function for estimating the target location u is

(36) $$L\left(S|u\right)=\prod_{n=1}^{N}\prod_{k=1}^{K}\frac{1}{\sqrt{2\pi }\sigma }\exp\left\{-\frac{1}{2\sigma^{2}}{\left(r_{n,k}-r_{0}+10\eta \ln∥u-s_{n}∥\right)}^{2}\right\},$$

where *S* is the amalgamation of the RSSI sample sets obtained from each AN, i.e., $S=S_{1}\cup S_{2}\cdots \cup S_{N}$.

To facilitate the subsequent derivation of the CRLB, it is necessary to define

(37) $$g_{n,k}\left(u\right)=\frac{1}{\sigma }\left(r_{n,k}-r_{0}+10\eta \ln∥u-s_{n}∥\right).$$

Obviously, $g_{n,k}\left(u\right)$ represents a standard Gaussian random variable, and the independence of $g_{n,k}\left(u\right)$ holds for different *n* and *k*. If $h\left(u\right)=\ln\left\{L\left(S|u\right)\right\}$ denotes the log-likelihood function, then

(38) $$h\left(u\right)=-NK\ln\left(\sqrt{2\pi }\sigma \right)-\frac{1}{2}\sum_{n=1}^{N}\sum_{k=1}^{K}g_{n,k}^{2}\left(u\right).$$

The element of the Fisher matrix J in the *i*-h row and the *j*th column is

(39) $${\left[J\right]}_{ij}=-E\left[\frac{\partial^{2}h\left(u\right)}{\partial {\left[u\right]}_{i}\partial {\left[u\right]}_{j}}\right],$$

where $E\left[\cdot \right]$ denotes the mathematical operation of calculating the expected value, i,j=1,2, ${\left[u\right]}_{1}=x$ and ${\left[u\right]}_{2}=y$. Then, we have

(40) $$\frac{\partial h\left(u\right)}{\partial x}=-\alpha \sum_{n=1}^{N}\sum_{k=1}^{K}g_{n,k}\left(u\right)\frac{x-x_{n}}{{∥u-s_{n}∥}^{2}},$$

(41) $$\frac{\partial h\left(u\right)}{\partial y}=-\alpha \sum_{n=1}^{N}\sum_{k=1}^{K}g_{n,k}\left(u\right)\frac{y-y_{n}}{{∥u-s_{n}∥}^{2}},$$

where α=10η/σ is a constant. The second derivative of $h\left(u\right)$ with respect to *x* is

(42) $$\frac{\partial^{2}h\left(u\right)}{\partial x^{2}}=-K\alpha^{2}\sum_{n=1}^{N}\frac{{\left(x-x_{n}\right)}^{2}}{{∥u-s_{n}∥}^{4}}-\alpha \sum_{n=1}^{N}\sum_{k=1}^{K}g_{n,k}\left(u\right)\frac{{\left(y-y_{n}\right)}^{2}-{\left(x-x_{n}\right)}^{2}}{{∥u-s_{n}∥}^{4}}.$$

The second term on the right side of Equation (42) represents a weighted sum of zero mean Gaussian random variables, thereby making it a zero mean Gaussian random variable as well. Hence,

(43) $${\left[J\right]}_{11}=-E\left[\frac{\partial^{2}h\left(u\right)}{\partial x^{2}}\right]=K\alpha^{2}\sum_{n=1}^{N}\frac{{\left(x-x_{n}\right)}^{2}}{{∥u-s_{n}∥}^{4}}.$$

Similarly, we can acquire

(44) $${\left[J\right]}_{22}=K\alpha^{2}\sum_{n=1}^{N}\frac{{\left(y-y_{n}\right)}^{2}}{{∥u-s_{n}∥}^{4}},$$

(45) $${\left[J\right]}_{12}={\left[J\right]}_{21}=K\alpha^{2}\sum_{n=1}^{N}\frac{\left(x-x_{n}\right)\left(y-y_{n}\right)}{{∥u-s_{n}∥}^{4}}.$$

By combining Equations (43)–(45), we can establish the relationship $J=K\alpha^{2}G$, which links the elements of G to J:

(46) $${\left[G\right]}_{ij}=\frac{1}{K\alpha^{2}}{\left[J\right]}_{ij}.$$

Generally, the deviation $∥\hat{u}-u∥$ is used to characterize the localization error in the WSNs, encompassing errors in both *x* and *y* directions. Therefore, the CRLB of the localization system can be determined:

(47) $$\mathrm{CRLB}=\sqrt{\mathrm{tr}\left(J^{-1}\right)}=\frac{1}{\sqrt{K}\alpha }\sqrt{\mathrm{tr}\left(G^{-1}\right)}.$$

As depicted in Equation (47), the CRLB of the localization system exhibits an inverse proportionality to the arithmetic square root of the number of APs within each AN. In other words, as *K* increases, the CRLB decreases accordingly. Therefore, leveraging AP clusters can effectively enhance the localization performance of the system.

## 8. Complexity Analysis

In addition to localization accuracy, the computational complexity should also be considered as the performance of localization methods. The increase in computational complexity not only results in higher energy consumption, but also leads to longer time consumption for localization, which subsequently affects the accuracy of tracking and localizing moving targets. In the following, we analyze the asymptotic complexity of the APCL method in its worst-case scenario. As the APCL method is composed of both RDW and ETL algorithms, their computational complexities can be calculated separately to obtain that of the entire APCL method.

The operations in the algorithm can be categorized into two groups: numerical and matrix operations. Numerical operations encompass addition, subtraction, multiplication, division, exponentiation, and comparison, while matrix operations involve matrix multiplication, inversion, eigenvalue decomposition, assignment and scalar multiplication. The computational complexity of the numerical operations is evidently O(1). $R^{p\times q}$ denotes a p×q dimensional matrix. If $Q\in R^{p\times q}$ and $G\in R^{q\times g}$ hold, then the multiplication of Q and G exhibits a computational complexity of O(p·q·g). In case $H\in R^{n\times n}$ holds, either inversion or eigenvalue decomposition for H demonstrates a computational complexity of $O(n^{3})$, while assignment or scalar multiplication of H has a computational complexity of $O(n^{2})$.

In the RDW algorithm, it can be found that each of the *N* ANs involved in localization performs operations from Step 2 to Step 6, as depicted in Algorithm Section 6. The operations executed at each step of the RDW algorithm and their corresponding complexities are presented in Table 2, the sum of the complexities of Steps 2 to 6 is O(11K+3). Considering that all *N* ANs need to perform these operations, the computational complexity of the RDW algorithm is O(11KN+3N).

The ETL algorithm, in contrast to the RDW algorithm, incorporates not only numerical operations but also matrix operations. The specific operations executed at each step of the ETL algorithm and their corresponding complexities are presented in Table 3. In summary, the complexity of the ETL algorithm is O(81N+319).

Therefore, the computational complexity of the RDW algorithm and the ETL algorithm combined is O(11KN+84N+319). To analyze the growth trend of the computational complexity of the APCL method on *K* and *N*, we consider only the highest-order term and ignore its constant coefficient, resulting in an asymptotic computational complexity of O(KN) for the APCL method.

## 9. Simulation Results

### 9.1. Simulation Setup

In this section, the performance of the APCL method is evaluated through Monte Carlo simulation experiments, utilizing MATLAB R2018b. It is assumed that the RSSI measured by each AP follows the log-normal model, represented by Equation (2). In this model, parameter $r_{0}$ is a constant determined by the performance of both transmitting and receiving devices in the system. Typically, this parameter is estimated by fitting a logarithmic curve to actual measured values, such as the reference value of −37.47 dBm mentioned in [24]. It should be noted that $r_{0}$ is measured prior to conducting experiments and not during online experimentation. Therefore, for simulation purposes, it may be appropriate to set $r_{0}=-40$ dBm. The path loss exponent, denoted as η, is assigned η=1.2 based on obstructed scenarios in [25]. σ represents the standard deviation of the shadow fading term and serves as a variable due to its relation to the fluctuation degree of RSSI. The range of σ is considered from 1 dBm to 6 dBm. The simulation includes multiple ANs, each consisting of a cluster of *K* APs. As mentioned in Section 3, these APs are distributed in a circular pattern centered around the AN, with a maximum radius of $R_{\max}$. By substituting η=1.2 into Equation (12), we can determine that $R_{\max}$ is equal to 4.16 cm.

As the APCL method comprises a cascade of RDW and ETL algorithms, each algorithm is analyzed separately. Whether for estimating the target RSSI or localizing the target, the corresponding estimation accuracy is dependent on the location of the target in the scene. The performance of the algorithm cannot be described by its performance at a single location alone; it must be evaluated using estimation results from a sufficient number of points in the scene. Therefore, the total root mean square error (TRMSE) is defined as

(48) $$\mathrm{TRMSE}=\frac{1}{M_{t}}\sum_{m=1}^{M_{t}}{\mathrm{RMSE}}_{m},$$

where $M_{t}$ is the number of distinct experimental locations for the target in the simulation scene, and ${\mathrm{RMSE}}_{m}$ denotes the RMSE obtained at each location. Therefore, TRMSE can serve as a reliable metric to assess algorithmic performance in estimating the entire scene.

### 9.2. Comparison of RSSI Estimation Algorithms for Target

**Figure 3:**
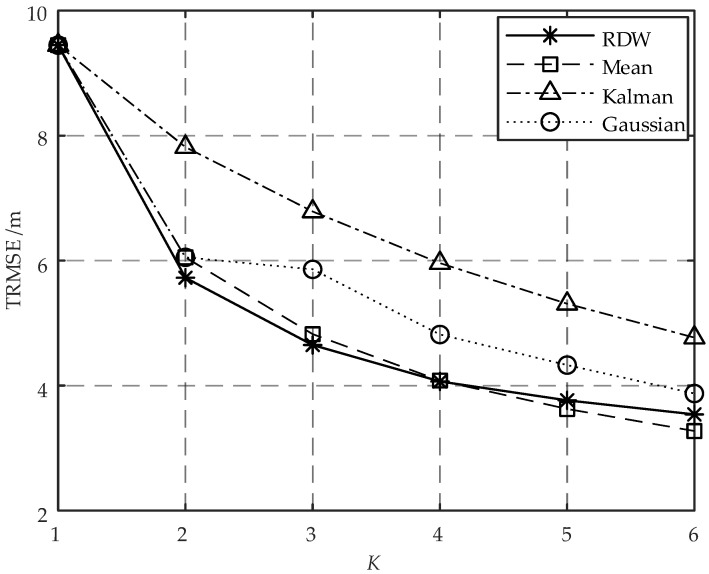
RSSI-based ranging error trend of different RSSI estimation algorithms with *K*.

To assess the accuracy of the RDW algorithm in estimating the RSSI of the target, a comparative analysis was conducted with conventional algorithms, including mean filter [14], Kalman filter [15], and Gaussian filter [16]. The value of σ was set to 6 dBm in order to represent a more severe fluctuation in RSSI. The location vector of AN is ${\left[0,0\right]}^{T}$, and the *K* APs constituting AN were distributed in a circular pattern centered around ${\left[0,0\right]}^{T}$ with a radius of 4 cm. The target was deployed at intervals of 1 m within a distance range of 1 m to 30 m from this AN location on the x-axis. To estimate the target RSSI for ranging, 1000 Monte Carlo experiments were conducted at each deployment location. The RMSE of ranging was calculated for each location. By combining the RMSE values obtained from all 30 deployment locations using Equation (48), the TRMSE was determined. The curves in Figure 3 illustrate the variation of TRMSE across different values of *K* for various RSSI estimation algorithms.

As depicted in Figure 3, at K=1, all algorithms exhibit equivalent TRMSEs since they degrade to RSSI estimation using a single sample. At K>1, the TRMSEs of all algorithms decrease with an increase in *K*, with exceptional performance observed for the RDW algorithm and the mean filter. Specifically, the RDW algorithm outperforms the mean filter for a small sample size (i.e., K=2 or 3). As the number of samples increases, the mean filter gradually demonstrates its advantages while exhibiting performance comparable to that of the RDW algorithm. The analysis of Figure 3 reveals that as *K* increases, the diminishing trend of TRMSEs for different algorithms gradually attenuates, indicating a reduced impact of *K* on enhancing ranging accuracy. Considering limitations in cost and space, it is advisable to opt for K=2 or 3.

### 9.3. Evaluation of Localization Algorithms

To assess the efficacy of the ETL algorithm and the APCL method, which comprises the ETL algorithm and the RDW algorithm, in estimating target location, they were compared with conventional localization algorithms including ${\mathrm{SDP}}_{\mathrm{RSS}}$ [17], SOCP1 [20], WLS [22], and SRLS [23]. Both the ${\mathrm{SDP}}_{\mathrm{RSS}}$ algorithm and the SOCP1 algorithm utilized the MATLAB package CVX with SeDuMi [27] as a solver to solve convex optimization problems.

The simulation localization scene is defined as a 20 m × 20 m area, with the lower left corner serving as the origin and arranging ANs at $s_{1}={\left[1,1\right]}^{T}$, $s_{2}={\left[1,19\right]}^{T}$, $s_{3}={\left[19,19\right]}^{T}$, and $s_{4}={\left[19,1\right]}^{T}$, respectively. Each AN is a cluster consisting of K=2 APs, and the *K* APs that make up AN are distributed in a circular pattern centered around the AN with a radius of 4 cm. In the simulation scenario, the target is deployed at intervals of 2 m × 2 m; then, the target can be deployed at most 121 different locations in the scenario, i.e., $M_{t}=121$ in Equation (48). The RMSE at each location is calculated by performing 50 Monte Carlo localization experiments, and the corresponding algorithm’s TRMSE is computed using Equation (48). Total Cramer–Rao lower-bound (TCRLB) can also be defined as

(49) $$\mathrm{TCRLB}=\frac{1}{M_{t}}\sum_{m=1}^{M_{t}}{\mathrm{CRLB}}_{m}.$$

**Figure 4:**
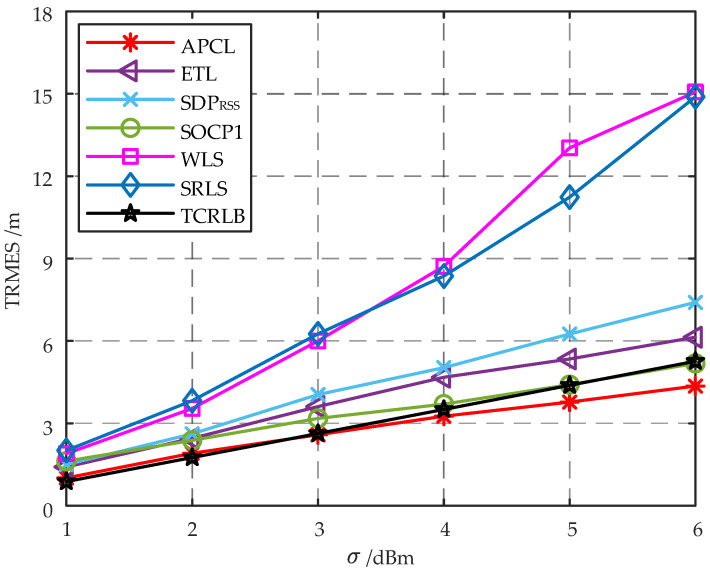
Localization error trend of different localization algorithms with σ.

The trend of TRMSE for different localization algorithms with σ is illustrated in Figure 4.

As shown in Figure 4, the TRMSEs of various algorithms exhibit an upward trend with increasing standard deviation of shadow fading σ increases. In terms of the same σ, both the WLS algorithm and the SRLS algorithm demonstrate higher TRMSEs compared to other algorithms. Furthermore, as σ increases, there is a significant increase in the TRMSEs for both the WLS and SRLS algorithms. Obviously, the inclusion of additional error components resulting from the approximation of the MLE problem by both the WLS and SRLS algorithms leads to a reduction in target localization accuracy. At lower σ, the TRMSEs of the ETL, ${\mathrm{SDP}}_{\mathrm{RSS}}$, and SOCP1 algorithms are nearly identical. However, as σ increases, there is an increasing divergence in TRMSEs among these three algorithms, with the SOCP1 algorithm exhibiting superior performance while the ${\mathrm{SDP}}_{\mathrm{RSS}}$ algorithm performs relatively worse. Therefore, the localization accuracy of the ETL algorithm is comparable to that of the convex relaxation method, disregarding the optimization of the target RSSI estimation. From Figure 4, it can be observed that the APCL method, which integrates the ETL algorithm with the RDW algorithm, exhibits superior localization accuracy. Since the APCL method yields a biased estimate of the target location, its TRMSE may be lower than the TCRLB when encountering higher-standard deviation of shadow fading σ. Similar findings have been documented in other studies, e.g., [13,17,20]. TCRLB represents the minimum achievable TRMSE for any unbiased estimator, while the APCL method exhibits a lower TRMSE than the TCRLB. This indicates that the target localization accuracy of the APCL method surpasses that of any unbiased estimator.

### 9.4. Comparison of Computational Complexity

The asymptotic computational complexity of the APCL method and several other typical target localization algorithms are presented in Table 4. It is evident that the WLS and SRLS algorithms exhibit significantly lower computational complexity compared to the ${\mathrm{SDP}}_{\mathrm{RSS}}$ and SOCP1 algorithms. Furthermore, the computational complexity of the APCL method is dependent not only on the number of ANs but also on the number of APs within an AP cluster. For the purpose of cost efficiency, the AP cluster typically consists of only 2 or 3 APs (i.e., K=2 or 3). In such cases, the computational complexity of the APCL method is comparable to that of WLS and SRLS algorithms.

**Figure 5:**
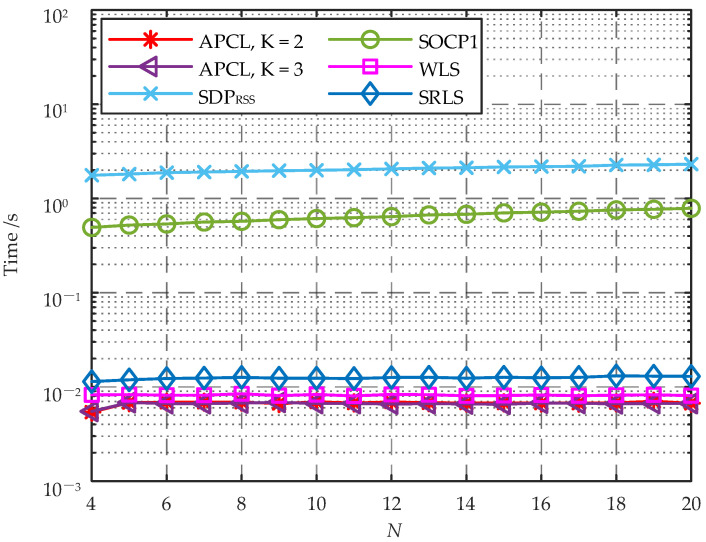
Average running time vs. number of ANs on an intel core i5-8300 H 2.30 GHz processor manufactured by Intel (Santa Clara, CA, USA).

To provide a more intuitive comparison of the computational complexity among different algorithms, we calculate the trend of the average running time with respect to *N* for each algorithm. The localization scenario used in simulation remains a 20 m × 20 m area with the target located at ${\left[10,10\right]}^{T}$, which is situated at the center of this area. Each AN utilized for localization comprises an AP cluster consisting of *K* APs, where K=2,3. The simulation environmental parameters are configured to $r_{0}=-40$ dBm, η=1.2 and σ=3 dBm. To analyze the average running time for target localization with varying numbers of ANs, 50 random layouts are performed in the simulation area for a given *N*. Different algorithms with different AN layouts are utilized to localize the target, and the average running time of each algorithm is calculated as shown in Figure 5.

As shown in Figure 5, the average running time for individual localization of ${\mathrm{SDP}}_{\mathrm{RSS}}$ and SOCP1 algorithms exhibits a significantly higher value compared to other methods. When K=2 or 3, the average localization time for the APCL method is marginally lower than those of the WLS and SRLS algorithms. The mean localization durations of APCL, WLS and SRLS algorithms remain relatively stable as *N* increases without any substantial changes, consistently within the millisecond range. These algorithms exhibit a lower computational complexity and are better suited for localization of moving targets.

## 10. Experimental Results

### 10.1. Experimental Setup

To further assess the performance of the APCL method, we conduct localization experiments in a real indoor environment. The experiment utilizes the Wi-Fi probe as an AP and a personal computer as the target. The Wi-Fi probes used are the DS006S and DS006N models manufactured by Chengdu Data Sky Technology Co., Ltd. (Chengdu, China). Both DS006S and DS006N have identical functional parameters, with the only distinction being that DS006S has external antennas while DS006N has internal antennas. The personal computer employed is the Lenovo Legion Y7000 notebook manufactured by Lenovo (Beijing, China), with the Windows 10 operating system. The carrier frequency for communication between APs and target is 2.4 GHz. During the data measurement, the APs monitor probe request frames broadcasted by the target, extracting MAC address and RSSI from these frames. The collected data from each AP are transmitted to the system processing center using the TCP protocol and processed for localization. The APs measure RSSI and transmit the data to the system processing center at a frequency of 1 Hz.

**Figure 6:**
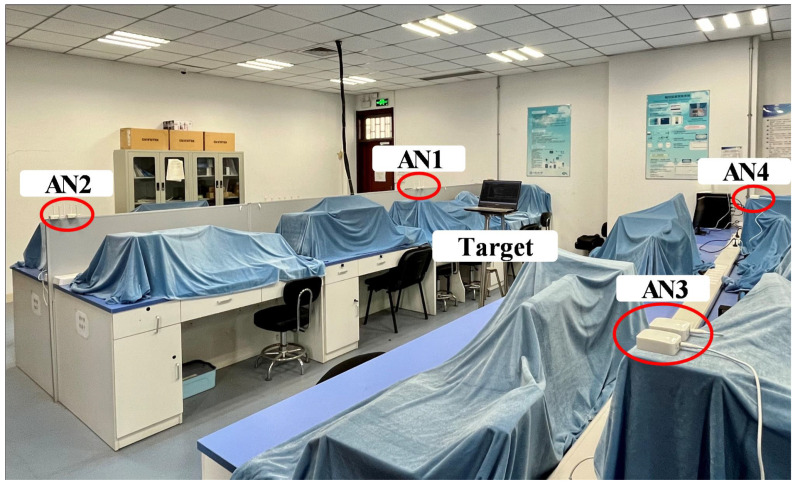
Indoor measurements environment.

The experimental scene is a room with dimensions of 11 m × 8.5 m × 3 m, where eight APs are deployed as depicted in Figure 6. Each pair of APs forms an AP cluster, constituting an AN, resulting in a total of four ANs. All APs are positioned on a 1.25 m high experimental table, and the target’s movement also takes place at a height of 1.25 m for the two-dimensional localization experiments. The RSSI samples of the target at various distances are premeasured within the scene, and the log-normal model parameters $r_{0}=-50$ dBm and η=0.76 in the environment are obtained through curve fitting functions in MATLAB. According to Equation (12), $R_{\max}=6.57$ cm can be obtained. In the subsequent experiment, we position each pair of APs comprising the AN in a circular pattern centered around the AN with a radius of 4 cm.

**Figure 7:**
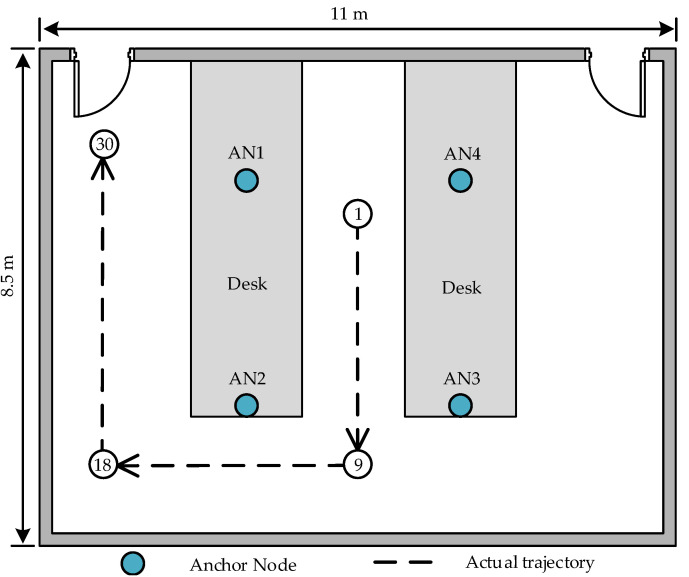
Floor plan of the experimental scene.

The position vectors of the four ANs are $s_{1}={\left[3.3,6.3\right]}^{T}$, $s_{2}={\left[3.3,2.1\right]}^{T}$, $s_{3}={\left[7.1,2.1\right]}^{T}$ and $s_{4}={\left[7.1,6.3\right]}^{T}$ respectively, while the position vectors of each AP are $s_{1,1}={\left[3.3,6.34\right]}^{T}$, $s_{1,2}={\left[3.3,6.26\right]}^{T}$, $s_{2,1}={\left[3.3,2.14\right]}^{T}$, $s_{2,2}={\left[3.3,2.06\right]}^{T}$, $s_{3,1}={\left[7.1,2.14\right]}^{T}$, $s_{3,2}={\left[7.1,2.06\right]}^{T}$, $s_{4,1}={\left[7.1,6.34\right]}^{T}$ and $s_{4,2}={\left[7.1,6.26\right]}^{T}$. The floor plan of our experimental setting is depicted in Figure 7, where the target follows a dashed trajectory from Point 1 to Point 30. Its position is recorded at every interval of 0.5 m along the track. There are a total of 30 sample points on the track, and Points 1 through 7 fall within the enclosed area formed by four ANs, as illustrated in Figure 7. The target RSSI is measured 50 times at each sample point by each AP, resulting in a test set of 3000 RSSI samples.

### 10.2. Localization Results

**Figure 8:**
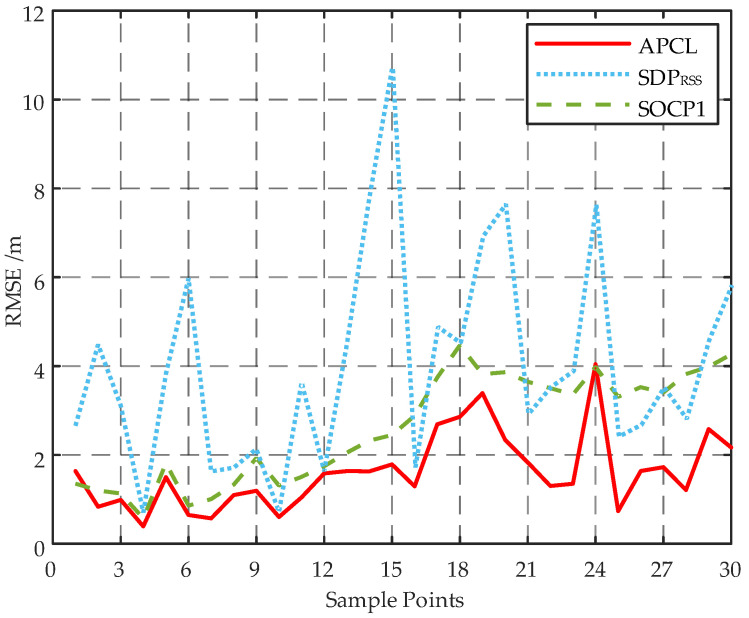
Localization error at different sample points of different localization algorithms.

To mitigate the impact of stochastic factors, a total of 50 localizations was conducted according to the samples in the test set for each sample point on the trajectory depicted in Figure 7. The resulting localization outcomes were utilized to calculate the RMSEs at various locations, as illustrated in Figure 8. Considering the inadequate precision exhibited by both the WLS and SRLS algorithms, as indicated in Figure 4, only the ${\mathrm{SDP}}_{\mathrm{RSS}}$ and SOCP1 algorithms are employed in Figure 8 for comparative analysis with the APCL method.

As shown in Figure 8, the RMSEs of the APCL method and the SOCP1 algorithm are comparable for the first seven points. However, for subsequent points, the RMSE of the SOCP1 algorithm is significantly higher than that of APCL method, indicating that APCL’s localization accuracy is less affected by the AN layout. Furthermore, at all points, the APCL method outperforms the ${\mathrm{SDP}}_{\mathrm{RSS}}$ algorithm in terms of RMSE.

The results in Table 5 demonstrate that the APCL method outperforms the ${\mathrm{SDP}}_{\mathrm{RSS}}$ and SOCP1 algorithms in terms of TRMSE, with a significantly lower value. Additionally, the APCL method exhibits a much shorter average running time compared to both ${\mathrm{SDP}}_{\mathrm{RSS}}$ and SOCP1 algorithms.

### 10.3. Localization Experiments Based on Public Datasets

**Figure 9:**
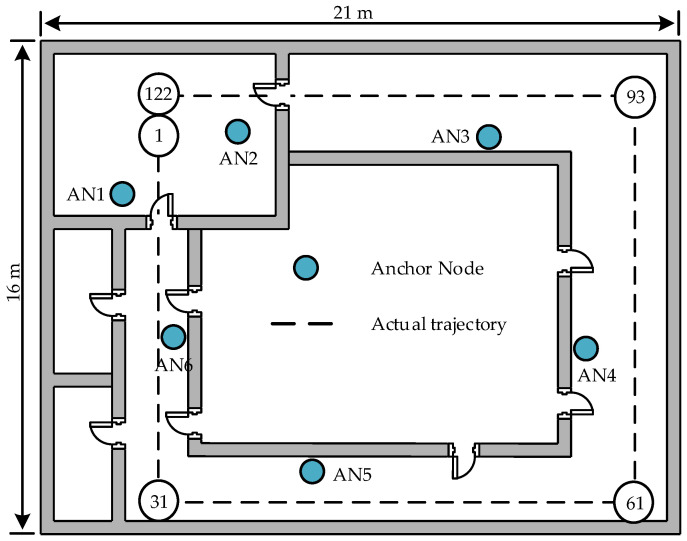
Floor plan of the experimental scene for the public dataset.

The localization performance of the APCL method in more complex scenes is further examined through experiments conducted using the public dataset [28]. Figure 9 illustrates the scene plan schematic of the public dataset, which has a size of 21 m × 16 m. The scene consists of six ANs, and the target follows a dashed trajectory from Point 1 to Point 122. The AN in the public dataset is composed of individual AP, whereas in this paper, the AN is composed of *K* APs. As discussed in Section 3, it is evident that the *K* APs forming the AN can be considered equivalent to a single AP with *K* samples. Hence, if each *K* sample of the AN in the public dataset is treated as a singular measurement, it can be regarded as composed of *K* APs.

**Figure 10:**
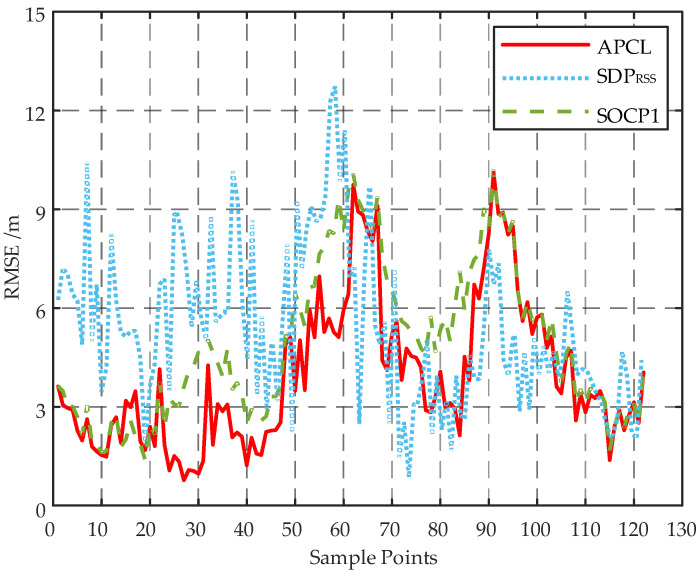
Localization errors on public dataset.

The 122 sample points in Figure 9 were individually localized 25 times according to the public dataset, and subsequently, the RMSEs were computed for each point based on the localization results, as illustrated in Figure 10.

The target is in close proximity to several ANs from Point 1 to Point 31, as depicted in Figure 10. Both the APCL method and the SOCP1 algorithm exhibit small RMSEs during this interval. From Point 31 to Point 61, the target initially approaches AN5 and then moves away from it, while also becoming more distant from other ANs. Consequently, the RMSEs of various methods show a decreasing trend followed by an increasing trend. The results from Point 61 to Point 93 exhibit a similar trend as those from Point 31 to Point 61. Moving from Point 93 to Point 122, the target gradually shifts towards an area with dense ANs, leading to a gradual decrease in the RMSEs of different methods. Across various locations, the APCL method consistently demonstrates lower RMSEs compared to the ${\mathrm{SDP}}_{\mathrm{RSS}}$ and SOCP1 algorithms. The TRMSEs of the APCL, ${\mathrm{SDP}}_{\mathrm{RSS}}$, and SOCP1 are 3.9438 m, 5.4709 m, and 4.8937 m, respectively. Consequently, it can be concluded that the APCL method exhibits superior localization accuracy compared to the other two algorithms.

## 11. Conclusions

The APCL method proposed in this paper is a technique for achieving active target localization using a WSN constructed by the AP clusters. The APCL method comprises the RDW algorithm for estimating RSSI of the target, followed by the ETL algorithm for localization. The RDW algorithm utilizes target RSSI samples acquired from the AP cluster to estimate the optimal RSSI of the target. In scenarios with limited sample sizes, the RDW algorithm exhibits superior advantages and achieves significantly higher accuracy in estimating the target RSSI compared to mean filter, Kalman filter, and Gaussian filter. The ETL algorithm transforms the MLE problem into an eigenvalue problem by constructing the eigenvalue equation using the estimated RSSI of each AN. The approach enables fast and accurate estimation of the target position. The positioning accuracy of the ETL algorithm is comparable to that of convex relaxation method while surpassing WLS and SRLS algorithms. The APCL method, consisting of the RDW and ETL algorithms, exhibits superior positioning accuracy, minimal positioning time, and low computational complexity. In the actual indoor positioning scenarios, the APCL method demonstrates more stable performance. Consequently, when compared to classical localization algorithms, the APCL method demonstrates significantly superior performance in terms of both positioning accuracy and average positioning time. Its high precision and efficient localization make it particularly suitable for indoor mobile target tracking.

The proposed method effectively reduces the computational effort of localization and achieves high accuracy. However, it necessitates prior knowledge of the parameters in the log-normal model for the localization scene. Further research is warranted to explore how to extend this paper’s method to target localization when environmental parameters are unknown. In addition, the proposed method assumes that all APs within the AP cluster possess identical hardware parameters. However, further research is required to address device heterogeneity and enhance the applicability of this approach.

## Figures and Tables

**Table 1:** List of Key Notations.

Notation	Explanation	Notation	Explanation
*N*	Number of the ANs	*K*	Number of the APs in an AP cluster
$s_{n}$	Location of the *n*th AN	u	Location of the target
$\hat{u}$	Estimated location of the target	$d_{n}$	Distance between the *n*th AN and the target
${\hat{d}}_{n}$	Estimated distance between the *n*th AN and the target	$r_{n,k}$	RSSI measured by the *k*th AP in the *n*th AN
${\hat{r}}_{n,\mathrm{opt}}$	RSSI estimated by the *n*th AN	$r_{0}$	RSSI at a distance of 1 m
η	Path loss exponent	$\sigma^{2}$	Variance of the shadow fading term

**Table 2:** Operations and complexities associated with the RDW algorithm.

Step No.	Operations Involved	Complexity
Step 2	K−1 additions and 1 division operation to derive ${\bar{r}}_{n}$; 1 subtraction, 2 divisions and 1 exponentiation to obtain ${\bar{d}}_{n}$	O(K+4)
Step 3	*K* subtractions, 2K divisions and *K* exponentiations to obtain ${\left\{{\bar{d}}_{n,k}\right\}}_{k=1}^{K}$	O(4K)
Step 4	*K* comparisons, *K* subtractions and *K* divisions to obtain ${\left\{\gamma_{n,k}\right\}}_{k=1}^{K}$	O(3K)
Step 5	K−1 additions to obtain $\gamma_{n}$	O(K−1)
Step 6	K−1 additions, *K* multiplications and 1 division to obtain ${\hat{r}}_{n,\mathrm{opt}}$	O(2K)

**Table 3:** Operations and complexities associated with the ETL algorithm.

Step No.	Operations Involved	Complexity
Step 1	*N* subtractions, 2N divisions and *N* exponentiations to obtain ${\left\{{\hat{d}}_{n}\right\}}_{n=1}^{N}$	O(4N)
Step 2	N−1 additions are required to obtain *w* and *N* divisions to acquire ${\left\{w_{n}\right\}}_{n=1}^{N}$	O(2N−1)
Step 3	2N multiplications and 2(N−1) additions to obtain $s_{w}$	O(4N−2)
Step 4	2N subtractions to obtain ${\left\{s_{n}^{\prime }\right\}}_{n=1}^{N}$	O(2N)
Step 5	13N multiplications and 3N additions to derive A; 6N multiplications and 2N additions to obtain $b^{\prime }$	O(24N)
Step 6	Eigenvalue decomposition on A with $A\in R^{2\times 2}$	O(8)
Step 7	Multiplication of matrix U with matrix $b^{\prime }$ to obtain b with $U\in R^{2\times 2}$ and $b^{\prime }\in R^{2\times 1}$	O(4)
Step 8	Construction of M with $M\in R^{5\times 5}$	O(25)
Step 9	Eigenvalue decomposition on M	O(125)
Step 10	For each *m*, 3 multiplications and 1 scalar multiplication of matrix, 1 division and 2 additions to obtain ${\hat{u}}_{m}$	O(160)
Step 11	For each *m*, 3 subtractions, 5 multiplications and 1 addition to obtain $C({\hat{u}}_{m})$	O(45N)

**Table 4:** Comparison of asymptotic computational complexity.

Algorithm	Description	Complexity
APCL	The proposed method	$O\left(KN\right)$
${\mathrm{SDP}}_{\mathrm{RSS}}$	The SDP estimator in [17]	$O\left(N^{4.5}\right)$
SOCP1	The SOCP estimator in [20]	$O\left(N^{3.5}\right)$
WLS	The WLS estimator in [22]	$O\left(N\right)$
SRLS	The localization method in [23]	$O\left(N\right)$

**Table 5:** TRMSE and running time of different algorithms on an intel core i5-8300 H 2.30 GHz processor manufactured by Intel (Santa Clara, CA, USA).

Algorithm	TRMSE/m	Average Running Time/s
APCL	1.6104	0.0083
${\mathrm{SDP}}_{\mathrm{RSS}}$	4.0264	1.7703
SOCP1	2.6058	0.4836

## Data Availability

Not applicable.

## Abbreviations

The following abbreviations are used in this manuscript:

RFID	Radio frequency identification
UWB	Ultra-wideband
LoRa	Long-range radio
BLE	Bluetooth low energy
TOA	Time of arrival
TDOA	Time difference of arrival
AOA	Angle of arrival
RSSI	Received signal strength indication
MLE	Maximum-likelihood estimation
APCL	Access point clusters localization
AP	Access point
WSN	Wireless sensor network
AN	Anchor node
SDP	Semidefinite programming
SOCP	Second-order cone programming
WLS	Weighted least squares
SRLS	Squared range least squares
RDW	Relative distance weighting
ETL	Eigenvector-based target localization
TCRLB	Total Cramer–Rao lower bound
TRMSE	Total root mean square error
